# Draft Genome Sequence of *Ruminoclostridium* sp. Ne3, Clostridia from an Enrichment Culture Obtained from Australian Subterranean Termite, *Nasutitermes exitiosus*

**DOI:** 10.1128/genomeA.00305-15

**Published:** 2015-04-23

**Authors:** Han Wang, Hai Lin, Nai Tran-Dinh, Dongmei Li, Paul Greenfield, David J. Midgley

**Affiliations:** a University of Science and Technology, Beijing, China; b CSIRO, Food & Nutrition Flagship, Dickson, Australia; c Plant Health & Environment Laboratory, Ministry for Primary Industries, Auckland, New Zealand; d CSIRO, Digital Productivity and Services, Dickson, Australia; e CSIRO, Energy Flagship, Dickson, Australia

## Abstract

The draft genome sequence of *Ruminoclostridium* sp. Ne3 was reconstructed from the metagenome of a hydrogenogenic microbial consortium growing on xylan. The organism is likely the primary hemicellulose degrader within the consortium.

## GENOME ANNOUNCEMENT

Global desire to use hydrogen as an energy source is growing as it is environmentally less damaging, greenhouse neutral, and has a high energy density (1, 2). Commercial production of hydrogen is mainly undertaken by physicochemical methods, though increasingly, biogenetically produced hydrogen is of interest due to its low cost and the potential to use waste streams as feedstock (3, 4). Biogenic hydrogen is produced as an end product of dark fermentation and is normally coproduced with some form of reduced carbon, typically CO_2_ (5). Termites naturally host prokaryotes that facilitate lignocelluloses digestion, including taxa that are known to produce hydrogen (6, 7). An enrichment culture, designated 1 TC, from the gut of a worker *Nasutitermes exitiosus* (collected, 33°45′34″ S; 150°59′25″E) was shown to convert xylan to commercially significant quantities of hydrogen with relatively little CO_2_ production.

The 1 TC culture was sequenced using Illumina HiSeq 2000, and resultant reads were assembled using Velvet 1.1.07. The metagenome was binned using short k-mer tools into genomes (8). In total, the metagenome encompassed three mostly complete clostridial genomes, two *Clostridiaceae sensu stricto* (9, 10) and one *Ruminococcaceae* genome. This manuscript describes the genome of the *Ruminococcaceae* taxon, *Ruminoclostridium* sp. Ne3. The closest completely sequenced relatives to Ne3 were *Ruminoclostridium cellulosi* (11) and *Ethanoligenens harbinense* (12). Based on 16S rRNA comparisons, undertaken separately using 16S PCR, and classification using RDP (13), Ne3 was most closely related to *R. cellulosi* (sequence, RDP: S001243254). Ne3 accounted for 12.9% of the metagenome and comprised 267 large (>200 bp) contigs which totaled ~2.6 Mbp. The mean, median, and *N*_50_ contig lengths were 10,023 bp, 8,233 bp, and 10,188 bp, respectively. The longest contig was 47,843 bp, and the G+C content was 54%. Annotation was performed using IMG-ER (Integrated Microbial Genomes Expert Review) (14), which predicted a total of 2,596 protein-coding genes and 36 structural RNAs. The annotated genome is available for download at IMG-ER (http://img.jgi.doe.gov/mer), and the sequences and metadata are available at the European Nucleotide Archive under accession no. PRJEB8629 (http://www.ebi.ac.uk/ena/data/view/PRJEB8629).

In order to understand how the 1 TC culture converts xylan to hydrogen, the carbohydrate active enzymes and hydrogenases that the component genomes possess were examined using the dbCAN (http://csbl.bmb.uga.edu/dbCAN/index.php) (15) and Pfam (http://pfam.xfam.org/) (16) online tools. It seems likely that *Ruminoclostridium* sp. Ne3 is the primary degrader of xylan in the consortium. Its genome encodes an array of catabolic genes including glycoside hydrolases from families implicated in activities against hemicellulose including various xylanases and xyloglucanases (GHs: 5, 8, 30, and 74), debranching enzymes (GH51, 67, 78, 106, and 127), and enzymes with activities against hemicelluloses-derived oligosaccharides (GH1, 2, 3, 39, and 105). Similar genes have been previously observed in termite microbiomes (17, 18). Along with carbohydrate active genes, genes involved in hydrogen production were also detected in Ne3, including two FeFe hydrogenases (PF02906, PF02256) and a NiFe hydrogenase (PF00374). Work to further characterize the genome of this taxon is ongoing.

### **Nucleotide sequence accession number**s. 

This whole-genome shotgun project has been deposited at DDBJ/EMBL/GenBank under the accession numbers CEMF01000001 through CEMF01000267.
